# How empowering leadership influences medical workers' work–family conflict in the post-pandemic era: A moderated mediation model of leadership “black box”

**DOI:** 10.3389/fpsyg.2022.870753

**Published:** 2022-07-28

**Authors:** Haiming Zhou, Xinping Song, Laitan Fang, Kan Shi, Ronghui Liu

**Affiliations:** ^1^Public Course Teaching Department of Shandong University of Science and Technology, Tai'an, China; ^2^ZR Holdings Limited, Beijing, China; ^3^Graduate School, Nueva Ecija University of Science and Technology, Cabanatuan, Philippines; ^4^Founder Technology College, Peking University, Beijing, China; ^5^School of Economic and Management, University of Chinese Academy of Sciences, Beijing, China; ^6^Wenzhou Model Development Institute, Wenzhou University, Wenzhou, China

**Keywords:** empowering leadership, role stress, work-family conflict, psychological detachment, medical workers, leadership “black box”

## Abstract

After experiencing the COVID-19 pandemic, the status and mechanisms of leadership, and the challenges for medical workers in terms of family–work conflicts, have caused widespread concern. In the post-pandemic era, based on role theory and the stressor-detachment model, this paper seeks to break the “black box” of negative effects that can be caused by leadership, research the mechanism and boundary conditions of those negative effects, and explore factors to reduce those negative effects. We recruited 1,010 Chinese medical workers fighting COVID-19 on the frontline. Our study results showed that there was a significant negative correlation between empowering leadership and work–family conflict, and this relationship was completely mediated by role stress, while psychological detachment moderated the relationship between role stress and work–family conflict. Moreover, psychological detachment moderated the mediating effect of empowering leadership on work–family conflict through role stress. Therefore, higher levels of psychological detachment were less conducive to medical workers' family–work conflict. This study has important theoretical significance and practical value for revealing the negative effects and mechanisms of empowering leadership and for medical workers to better deal with work–family relations.

## Introduction

In 2020, COVID-19 swept the world. In the face of this pandemic, there was a run on medical resources, and medical workers were in short supply. Countries were forced to adopt relatively policies limiting public freedoms, and organizations were facing deepening uncertainty and often heading into crisis. At the same time, the flattening of organizational structures and the creation of mobile offices became more common, and leaders and employees had to rush to the front line, which caused many leaders and employees to fall into a “feedback vacuum” (Zhang J. et al., 2020). In such an environment, top-down leaders—characterized by a command and control approach—have found it difficult to adapt to, and dynamically match, the environment they face; in addition, the significance of the alternative “empowering type” of leadership is becoming increasingly prominent (Qian et al., 2018). Empowering leadership refers to the leadership style that motivates employees' self-management, self-leadership, and participation in goal setting (Pearce et al., 2003). Giving employees the opportunity to participate in decision-making, this leadership style stimulates the internal motivation of employees, affecting working attitudes and behavior. Many researchers have shown that empowering leadership can improve employees' job satisfaction (Kim et al., 2018), job performance (Harris et al., 2014), and innovative behavior (Amundsen and Martinsen, 2015). However, the research shows that empowering leadership is not always a positive influence (Wang and Sun, 2019). In the workplace, empowering leadership may also have negative effects, such as reducing employees' work efficiency, job performance, job satisfaction, and job wellbeing (Bamberger et al., 2017). At present, in the doctor–patient domain of China, a series of malignant medical injuries has a negative impact on the psychology of medical workers and their families; however, their tense living and working environment does not affect medical workers' selfless dedication to the “wartime” fighting of the pandemic; we thank these medical workers for making great contributions and indeed sacrifices at this time (Wang, 2020). In turn, we should pay more attention to them and care for them, so they can carry on the fight more calmly, without being so heavily affected by family concerns. Work–family conflict is still a strong research issue (Gao and Zhao, 2014; Ma et al., 2014; Chen et al., 2017; Jia and Su, 2020). In the post-pandemic era, COVID-19, albeit mutated, remains with us, changing our lives. Thus, the importance, stability, and sustainability of leadership and medical workers continue to be prominent.

In addition to the medical workers themselves, the negative effects of medical workers' leadership, work–family conflict, and mental health are worthy of research (Cheong et al., 2016; Chen et al., 2020b; Kang et al., 2020; Vaziri et al., 2020; Xiang et al., 2020; Zhang W. R. et al., 2020; Zhu et al., 2020; López-Núez et al., 2021; Lv et al., 2021), in particular to mitigate the negative effects of the so-called leadership “black box,” and to study interventions to work–family conflicts (Luo et al., 2007; Jin et al., 2014), highlight the positive effects of leadership and reduce the negative effects of leadership (Lee et al., 2017; Lin and Luo, 2017; Hao et al., 2018; Wong and Giessner, 2018; Yin and Xing, 2018; Wang and Sun, 2019; Chen et al., 2020a; Jiang and Xu, 2020; Song and Chen, 2021). Among the factors influencing work–family conflict, previous study has ignored the role of leadership behaviors (Li, 2018). Some leadership behaviors can overcome work–family conflict through the improvement of employee abilities (Bianchi and Milkie, 2010), while other leadership behaviors cannot meet work and family needs of employees at the same time, leading to work–family conflict (Beauregard, 2011). A recent review revealed that empowering leadership had a significant influence on employees' work–family conflicts (Wang and Sun, 2019). Empowering leadership gives employees the right to self-decision-making, trusting that subordinates can take on responsibilities and duties, and in turn better complete their work tasks. However, in the specific implementation process, employees do not understand the true intention of empowering leadership. At the same time, it is hard to meet the expectations and requirements of leaders, and empowering leadership leads to greater role pressure. This increase in job pressure affects the efficiency with which employees complete tasks. Their work rhythm is affected, and it is difficult to complete tasks within the specified timeframe, ultimately generating work–family conflict.

In addition to exploring the mediating role of job role stress, this paper also explores the moderating role of psychological detachment between empowering leadership and work–family conflict. Psychological detachment is a subjective experience that reflects employees' detachment from work at the psychological level (Sonnentag and Fritz, 2015). Psychological detachment is one important way for employees to recover after work, helping them regain physical and psychological “energy” and maintain a positive working mood (Johannes and Andrea, 2017; Ma et al., 2021). At work, when employees experience work stress and cannot eschew their psychological detachment, they become tired and thus less able to take the initiative. Therefore, their ability or otherwise to switch off psychological detachment has an important impact on their subsequent work behavior (Shi and Zhen, 2021). Some studies found that work–family conflict caused by role stress has different effects dependent on differences in employees' psychological detachment. Those employees who can remove psychological detachment can more effectively distinguish between work and life, find it easier to invest in family life, and thus experience less work–family conflict, while other employees are unable to eliminate psychological detachment, who have unclear boundaries between work and non-work. They thus are more inclined to “take work home” and cannot switch roles between work and family, leading to experience more work–family conflict. Increases in role conversion intensify the conflict between employees' roles, resulting in work affecting family life resulting in work–family conflict (Desrochers et al., 2005; Wang et al., 2021). Therefore, employees' psychological detachment plays an important regulatory role.

Based on the above analysis, this investigation studied medical workers, especially those in the frontline of fighting the pandemic. These workers are deeply affected by the cruelty of the pandemic and the tragic situation of many COVID-19 patients. The pandemic is potentially having an impact on their physical and mental health (Chen et al., 2020b; Kang et al., 2020; Lai et al., 2020; Simione and Gnagnarella, 2020; Xiang et al., 2020; Zhang W. R. et al., 2020; Zhu et al., 2020), in turn affecting their work and family relations (Vaziri et al., 2020; López-Núez et al., 2021; Lv et al., 2021). Focusing on this special group of workers, this paper explored the impact of empowering leadership on employees' work–family conflicts and analyzed the mediating effect of role stress and the moderating effect of psychological detachment from the perspective of role theory and the stressor-detachment model. In the post-pandemic era, it is not only of practical significance but also of great theoretical value to break the negative effect of the leadership “black box” and explore the potential impact of leadership behavior on the working state of employees, so as to provide a theoretical basis for positive interventions in management.

## Theory and hypotheses

### Empowering leadership and work–family conflict

There are two main perspectives in the study of empowering leadership. The first perspective is structural empowerment, which emphasizes the behavior process of leaders' empowerment and responsibility to employees, and mainly focuses on how leaders make empowerment decisions and how to implement specific strategies of empowerment (Leach et al., 2003). The second perspective is psychological empowerment, which focuses on employees' subjective perception and evaluation of leadership empowerment, and the empowerment under this perspective includes four dimensions: job meaning, competence, autonomy, and influence (Wang and Sun, 2019). From the perspective of psychological empowerment, empowering leadership advocates that employees should become self-leaders. By giving employees the power of self-control and self-decision-making, they can enhance their initiative and self-management ability. The structural dimension of empowering leadership itself includes the dimension of autonomy, which is an important component and element of empowering leadership; in addition, the effectiveness of empowering leadership includes the autonomy that affects subordinates (Vecchio et al., 2010; Hao et al., 2014; Schilpzand et al., 2018). Therefore, we conduct this study of empowering leadership from the perspective of psychological empowerment. Empowering leadership advocates that employees' ability of self-management and self-leadership can be stimulated through power sharing between them (Lee et al., 2017). This process of empowerment reflects the process of power transmission from leaders to subordinates and so is called “super leadership” (Manz and Sims, 1991). In addition, empowering leaders give employees the power of self-control and self-decision-making. After empowerment, this form of leadership also implies the leaders' expectation of taking responsibility for their subordinates, and a show of their confidence and expectation that their subordinates will perform highly (Hao et al., 2018). Moreover, empowering leaders encourage employees to participate in the decision-making of the organization. In the process of participating in decision-making, how to clarify the responsibilities and obligations of leaders and subordinates, especially how to reach agreement on potential responsibilities and obligations, is the key to removing the role of ambiguity for employees. In real work, if employees do not meet the high standards expected of them subsequent to leadership empowerment, and if subordinates do not clearly understand how to use empowerment to achieve leadership goals, employee leadership effectiveness and employee performance may be reduced (Humborstad and Kuvaas, 2013). This double-edged sword of empowering leadership has gained considerable attention within academic circles: empowering leadership not only brings positive effects but also has certain negative effects at the individual, team, and organizational levels (Lee et al., 2017; Lin and Luo, 2017; Yin and Xing, 2018).

In the field of empirical research, Jiang and Xu (2020) investigated employees of high-tech enterprises in China's Yangtze River Delta in terms of the negative effects on them of empowering leadership. Their study found that empowering leadership has a “too much rather than too little” effect on employees' task performance. When empowering leadership rises from a medium level to high level, employees' performance at their tasks decreases. In addition, Song and Chen (2021) found that the mismatch between subordinates' needs and accepted empowering leaders will lead to subordinates' emotional exhaustion. Compared with insufficient empowerment, if leaders over empower their staff, then those staff will experience emotional exhaustion. This negative effect of empowering leadership is also reflected in the work–family conflict of employees. On the one hand, this is because the self-leadership advocated by empowering leadership increases the uncertainty of work and, on top of the original task, additional tasks are added, resulting in excessive workloads and increased work ambiguity for employees (Humborstad and Kuvaas, 2013; Lorinkova et al., 2013; Wong and Giessner, 2018; Chen et al., 2020a). Based on the spillover effect, the pressure of work exerted on employees is bound to affect their family life, resulting in work–family conflict (Lin and Ling, 2016; Hao et al., 2018). On the other hand, according to the dual-task processing effect, employees tend to undertake multiple tasks at the same time. The spreading of effort between multiple tasks can weaken the personal resources of the workers. The loss of those resources makes it difficult for employees to cope with the demands of family, leading to conflicts between work and the family (Cheong et al., 2016; Wang and Sun, 2019). Empirical work shows that empowering leadership can lead to negative role definitions for employees, and this negative role definition can have an impact on aspects of work-related stress, also generating work–family conflict (Fong and Snape, 2015). Based on the above theoretical analysis and empirical findings, this paper proposes Hypothesis 1: Empowering leadership is negatively related to work–family conflict.

### The mediating role of role stress

Role theory is an important source of explanation about the mechanism underpinning the negative effects of empowering leadership (Cheong et al., 2016; Schilpzand et al., 2018). Role theory points out that roles include the cognition of its own roles, and the expectations and requirements of society for their own roles. In the process of engaging in their own role requirements, if the external role requirements extend beyond a certain limit, or the boundary is not clear, pressure increases on a person's work, that is, role stress is generated. Role overload, role ambiguity, and role conflict are three important sources of role stress (Dasgupta, 2012). Role stress often occurs in healthcare workers (Leiter and Maslach, 1988; Bakker et al., 2000; Garrett and McDaniel, 2001), where heavy workloads and the death of patients are the two major sources of stress for nurses (Hipwell et al., 1989). Antecedents of work–family conflict can be within the same work or family domain, or across domains (Creary and Gordon, 2016). Role overload exists when an individual fulfills multiple roles simultaneously and lacks the resources to perform them, which can result from both excessive time demands and excessive psychological demands (Creary and Gordon, 2016). Role ambiguity refers to the fact that employees do not understand the expectations of role givers for their specific roles due to personal reasons or external influences or lack of accurate expectations about the results of their behaviors. Empowering leadership increases the risk of employees' role ambiguity. When empowering leaders delegate tasks to subordinates, they expect more subordinates to be able to solve work-based problems independently, rather than having to explain to those subordinates exactly how to complete the task. This greatly increases the uncertainty for subordinates undertaking the work, leading to the risk of role ambiguity (Lorinkova et al., 2013). Previous research has pointed out that during time at work, empowering leaders tend to overestimate the ability of subordinates and believe that if authority over the task was delegated to subordinates, subordinates would complete the task according to their own expectations. However, it was found that subordinates could not predict their role goals and responsibilities, which led to further strengthening of role ambiguity (Humborstad and Kuvaas, 2013). Role conflict refers to incongruent expectations, which can occur both between and within roles (Schaubroeck et al., 1989). Role conflict occurs when two or more social roles overlap and are incompatible, and because the performance of one role interferes with the performance of another; this can be time-based, strain-based, or behavior-based (Creary and Gordon, 2016). Empowering leadership leads to role conflict in subordinates (Humborstad and Kuvaas, 2013; Song and Chen, 2021). First, after accepting the empowerment of leadership, subordinates face new roles in addition to dealing with the existing roles. The inconsistency between these two roles leads to role conflict. Furthermore, after empowering subordinates, the duties of subordinates increase, and these new responsibilities conflict with their existing roles, increasing role conflict. Role conflicts also arise when individuals are unable to reconcile the problems faced by various roles or when they are faced with conflicting expectations of roles transmitted by parties within and outside the organization (Tushman, 1978). Whether role conflict or role ambiguity, these issues consume resources between conversions in different domains, and this inter-occupancy of resources exists in both the work and family domains. Specifically, when people perceive role stress in a certain domain, they redistribute their resources among their different roles to attain resource transfer across domains. However, the resources of employees are limited, and thus resource use in one domain takes resources from another domain, leading to conflict between the two domains (Lazarus, 2001). When employees' resources in one domain are insufficient to accomplish their tasks, they will use resources originally allocated to another domain, leading to conflicts (Matthews et al., 2014). The process of coping with this stress also consumes their finite resources. Under the condition of limited resources in the existing domain of work, it is bound to crowd out resources in the family domain and eventually lead to conflicts between working life and family life (Matthews et al., 2014). Consequently, this paper proposes Hypothesis 2: Role stress plays a mediating role between empowering leadership and work–family conflict.

### The moderating role of psychological detachment

Psychological detachment refers to the phenomenon that individuals are freed from work-related affairs during non-working time and are no longer disturbed by them (Sonnentag et al., 2013). Psychological detachment can alleviate the influence of work stress on employees' work–family conflicts. According to the stressor-detachment model, psychological detachment buffers between stressors and other outcome variables such as nervousness. Psychological detachment as a potential buffer can alleviate the negative influence of stressors on nervous reactions, physical and mental symptoms, and health satisfaction (Sonnentag, 2011). After leaving the workplace and returning home, higher psychological detachment blocks the further depletion of resources caused by work stress, enabling individuals to accumulate psychological resources and providing adequate resources for subsequent work. For example, psychological detachment buffered the impact of workplace bullying on psychological tension (Moreno-Jiménez et al., 2009); in a follow-up study, psychological detachment moderated the impact of job demands on physical and mental symptoms and work engagement (Sonnentag and Bayer, 2005). In a diary study, the relationship between psychological disengagement, mobile phone use, and employees' life satisfaction was discussed. The results showed that resource supplementing brought by psychological disengagement had a positive impact on employees' life satisfaction (Wang et al., 2021).

Based on the above analysis, role stress, as a stressor, will weaken the impact on the work–family conflict because of the buffer effect of psychological detachment. Employees who can clearly divide work and non-work boundaries can achieve higher psychological detachment after work and stop thinking about work-related affairs, thus reducing the negative impact of role stress on their family life, in turn resulting in fewer work–family conflicts. However, those employees with lower psychological detachment will continue to undertake work-related affairs after leaving the workplace, intruding on family life, and resulting in work–family conflicts. Based on the above analysis, this paper proposes Hypothesis 3: Psychological detachment moderates the mediated relationship between empowering leadership and work–family conflict *via* role stress. Compared with employees with higher psychological detachment, the role stress of employees with lower psychological detachment has a greater predictive role on work–family conflict.

**Figure 1:**
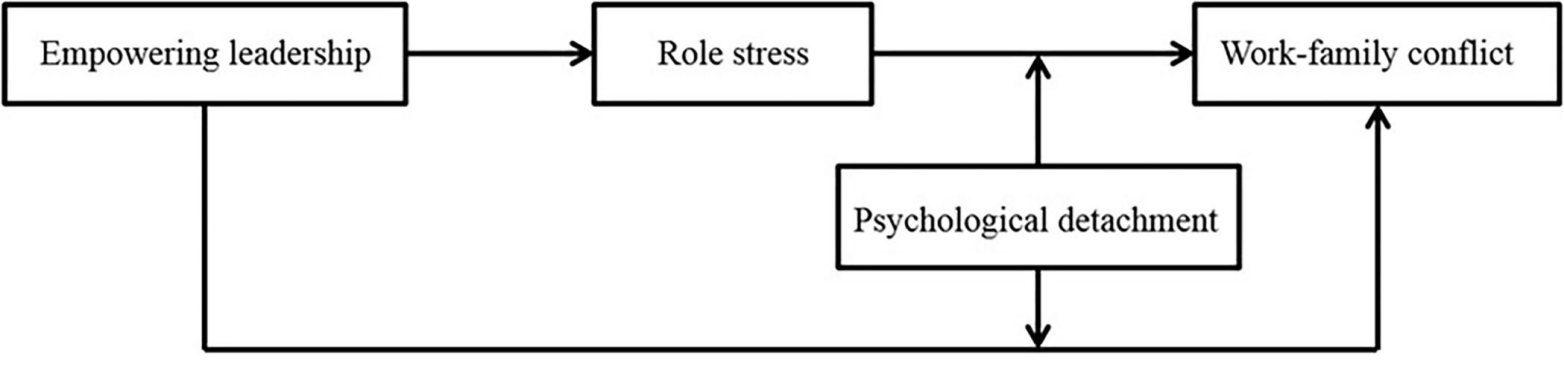
The moderated mediation model of leadership “black box”.

Based on the above framework, this study explored the impact of empowering leadership on work–family conflict in medical workers. This study analyzed the mediating role of role stress and the moderating role of psychological detachment from the perspective of role theory and stressor-detachment model, to establish a moderated mediation model for breaking the leadership “black box” of negative effects. The moderated mediation model of the leadership “black box” is shown in Figure 1.

## Materials and methods

### Participants

In this study, medical workers from several hospitals in the Shandong Province of China took part in a survey based on convenience sampling methods. This method was reviewed and approved by the morality and ethics committee of the Public Course Teaching Department of Shandong University of Science and Technology. The participants provided orally informed consent to this study. All 1,100 questionnaires were distributed, and 1,010 valid questionnaires were recovered, representing an effective return rate of 91.82%. Among the respondents, 160 were male (15.8%), and 850 were female (84.2%); 138 participants were below 25 years old (13.7%), 324 were between 26 and 35 years old (32.1%), 345 were between 36 and 45 years old (34.2%), and 203 were above 46 years old (20.1%). Of the respondents, 258 were unmarried (25.5%), 740 were married (73.3%), and 3 were divorced (1.2%); 179 were educated to lower than undergraduate degree level (17.7%), 671 had an undergraduate degree only (66.4%), and 160 had a postgraduate degree or higher (15.8%). Of the respondents, 885 were employees (87.6%), and 125 were managers (12.4%). The average and standard deviation of working years were 9.77 ± 9.11.

### Measures

#### Empowering leadership scale

The three-item empowering leadership scale (Schilpzand et al., 2018) was used to measure empowering leadership. Example items included “My leader made many decisions with me” and “My leader allowed me to finish my work in my own way.” All items were rated on a five-point Likert scale (1 = “strongly disagree” and 5 = “strongly agree”). In this paper, Cronbach's alpha for the scale was 0.87.

#### Work–family conflict scale

The ten-item Work–Family Conflict Scale (Netemeyer et al., 1996) was used to measure work–family conflict, which has two dimensions: work interference family and family interference work. Each dimension includes five questions. Example items included “The demands of my family or spouse/partner interfere with work-related activities” and “Family-related strain interferes with my ability to perform job-related duties.” All items were rated on a five-point Likert scale (1 = “strongly disagree” and 5 = “strongly agree”). Cronbach's alpha for the scale was 0.91, and Cronbach's alpha for both subscales was 0.94.

#### Role stress

The eight-item Role Stress Scale (Price, 2001) was used to measure role stress. Example items included “I know exactly what is expected of me in my job” and “I often get conflicting job requests from different supervisors.” All items were rated on a five-point Likert scale (1 = “strongly disagree” and 5 = “strongly agree”). In this paper, Cronbach's alpha for the scale was 0.80.

#### Psychological detachment scale

The four-item psychological detachment scale deriving from Sonnentag and Fritz's Recovery Experience Questionnaire (2007) was used to measure psychological detachment from work (Sonnentag and Fritz, 2007; Sonnentag et al., 2013). Example items included “During after-work hours I do not think about work at all” and “I forget about work.” All items were rated on a five-point Likert scale (1 = “strongly disagree” and 5 = “strongly agree”). Cronbach's alpha for the scale was 0.85.

### Control variables

According to previous studies, employees of different gender, age, and education tend to have different working and personal resources (Xanthopoulou et al., 2007). These resources directly influence people's experience of their role and their level of psychological detachment (Seibert et al., 2011; Bakker and Demerouti, 2017). Thus, gender, age, and education were included as dummy variables and control variables in this paper. In terms of gender, male was coded as “1” and female as “2.” For age, we set up three dummy variables: D1, D2, and D3. D1: ages below 25 years were coded as “1” and others as “0.” D2: 26–35 as “1” and others as “0.” D3: 36–45 as “1,” others as “0,” and above 46 both as “0.” For education, we set up two dummy variables: E1 and E2. E1: below a college degree was coded as “1” and others as “0.” E2: undergraduate as “1,” others as “0,” and postgraduate or higher both as “0.”

### Analysis

SPSS 20.0 was used for the statistical analyses [SA; including correlation analyses (CA) and linear regression analyses (LRA)]. To further test the moderated mediation effect, Process Model 4 and Amos 17.0 were used to apply the Bootstrap method (Hayes, 2013, 2017, 2018; Hair et al., 2019). To verify the moderated mediation model, the mediating and moderating effects were integrated into the same analytical framework (Wen et al., 2006; Cui and Li, 2021; Lv et al., 2021).

## Results

### Common method bias test

This paper used the Harman's single-factor test for the common method bias test (Podsakoff et al., 2003). The results revealed that the KMO value was 0.89 (*p* < 0.001), indicating that the scales were suitable for factor analysis. There were six factors with eigenvalues >1, and the first factor explained 30.12% of the variance, which was less than the critical criterion of 40%. Therefore, the impact of common method bias was not considered to be important (Fournier et al., 2021).

### Confirmatory factor analysis

To test the construct validity of the major variables, Amos 17.0 was used to conduct confirmatory factor analysis (CFA). Based on the method for testing the construct distinctiveness of the main variables (Wang et al., 2005), comparisons were made between the one-factor model (Model 1), the two-factor model (Model 2), the three-factor model (Model 3), and the four-factor model (Model 4). In Model 1, empowering leadership, role stress, psychological detachment, and work–family conflict were loaded on one factor. In Model 2, based on the previous research, empowering leadership and role stress were the items loaded on one factor, and psychological detachment and work–family conflict were the items loaded on another factor (Zeng and Yan, 2013; Hu and Wang, 2014). In Model 3, based on previous research, empowering leadership and role stress were the items loaded on one factor, and psychological detachment and work–family conflict were the items loaded on another factor, respectively (Hu and Wang, 2014; Zeng et al., 2018). In Model 4, the four constructs (empowering leadership, role stress, psychological detachment, and work–family conflict) were loaded as four independent factors. The results showed that Model 4 fitted the data better than did the other models and showed good construct validity (see Table 1).

**Table 1:** Confirmatory factor analysis to assess construct validity.

**Model**	**Factor loaded**	*χ^2^**/ df***	***df***	Δχ2/Δ*df*	**RMSEA**	**NFI**	**RFI**	**IFI**	**CFI**	**GFI**
Model 4	Four factors: EL, RS, PD, WFC	5.15	269	/	0.05	0.95	0.91	0.96	0.96	0.97
Model 3	Three factors: EL, RS, PD+WFC	9.09	272	452.38(3)^***^	0.10	0.86	0.89	0.86	0.86	0.91
Model 2	Two factors: EL+RS, PD+WFC	16.49	274	626.58(5)^***^	0.19	0.74	0.70	0.75	0.75	0.71
Model 1	One factor: EL+RS+PD+WFC	24.15	275	875.98(6)^***^	0.21	0.66	0.61	0.67	0.67	0.62

*WFC, work–family conflict; PD, psychological detachment; RS, role stress; EL, empowering leadership; +, the combination of two factors into one factor*.

*** *p < 0.001*.

### Descriptive statistics and correlation analysis

Among the variables, gender, age, and education background are virtualized. In terms of gender variable, women are taken as the standard, represented by 0, and men by 1. There are more than two categories of age and educational background variables, which are also dummy variables with values of 1 and 0. The specific operation steps are completed through the existing operation steps in SPSSAU. As shown in Table 2, the results of descriptive statistics and correlations among all variables revealed that empowering leadership was negatively related to role stress (*r* = −0.46, *p* < 0.01) and work–family conflict (*r* = −0.14, *p* < 0.01) and positively related to psychological detachment (*r* = 0.14, *p* < 0.05). The results also indicated that role stress was positively related to work–family conflict (*r* = 0.50, *p* < 0.01), and psychological detachment was negatively related to work–family conflict (*r* = −0.11, *p* < 0.05), supporting Hypothesis 1. However, there was no significant correlation between role stress and psychological detachment (*r* = 0.02, *p* > 0.05). These results provided the basis for testing the other study hypotheses.

**Table 2:** Descriptive statistics and correlations between the major variables.

**Variable**	***M***	***SD***	**1**	**2**	**3**	**4**	**5**
1. Work time	9.77	9.11	-				
2. Empowering leadership	2.98	1.15	0.00	(0.87)			
3. Role stress	2.24	0.63	−0.06	−0.46^**^	(0.80)		
4. Psychological detachment	2.97	0.89	0.02	0.14^*^	0.02	(0.85)	
5. Work–family conflict	2.65	0.82	−0.10^*^	−0.28^**^	0.50^**^	−0.11^*^	(0.91)

* *p < 0.05;*

** *p < 0.01*.

### The mediation role of role stress

First, the mediating model examined whether role stress mediated the relationship between empowering leadership and work–family conflict. Subsequently, this paper used the SPSS PROCESS-Model 14 to examine the mediating role (Hayes, 2013). Bootstrapping was used to generate confidence intervals for the indirect effects (Williams and MacKinnon, 2008). Mediation was established when the indirect effect was significant, and the confidence intervals did not contain zero. In Table 3, after controlling for demographics (gender, age, and educational level), the results revealed that empowering leadership negatively predicted work–family conflict [Model 2: *B* = −0.20, 95% CI (−0.24, −0.15), SE = 0.02, *t* = −8.45, *p* < 0.001]. After bringing empowering leadership and role stress together into the regression equation, the results showed that empowering leadership did not predicted work–family conflict [Model 3: *B* = −0.05, 95% CI (−0.10, 0.01), SE = 0.03, *t* = −1.85, *p* > 0.05], but role stress positively predicted work–family conflict [Model 3: *B* = 0.61, 95% CI (0.52, 0.71), SE = 0.04, *t* = 13.2, *p* < 0.001]. The results showed that role stress had a significant and complete mediating role in the relationship between empowering leadership and work–family conflict [completely standardized indirect effect = −0.22, 95% CI = (−0.26, −0.18)], the confidence interval not containing zero, supporting Hypothesis 2.

**Table 3:** Mediating effect of role stress on the relationship between empowering leadership and work–family conflict.

**Dependent variable**	**Model 1 (role stress)**		**Model 2 (Work–family conflict)**		**Model 3 (Work–family conflict)**		**Model 4 (Work–family conflict)**	
	***B***	***SE***	***B***	***SE***	***B***	***SE***	***B***	***SE***
Gender^a^	−0.10	0.04	−0.05	0.07	0.02	0.06	0.02	0.06
Age^b^								
D1	0.19	0.11	0.31^*^	0.15	0.20	0.14	0.23	0.14
D2	0.17	0.09	0.33^**^	0.13	0.23^*^	0.11	0.24^*^	0.11
D3	0.14	0.08	0.25^*^	0.11	0.17	0.10	0.18	0.10
Educational level^c^								
E1	−0.01	0.07	−0.18^*^	0.10	−0.17^*^	0.09	−0.13	0.08
E2	−0.04	0.05	−0.18^*^	0.08	−0.15^*^	0.07	−0.12	0.07
Work time	0.01	0.01	0.01	0.01	0.01	0.01	0.01	0.01
Empowering leadership	−0.25^***^	0.02	−0.20^***^	0.02	−0.05	0.02		
Role stress					0.61^***^	0.04	0.17	0.12
Psychological detachment							−0.44^***^	0.08
Psychological detachment * role stress							0.16^***^	0.04
*R^2^*	0.22	0.10	0.27	0.29				
*F*	35.78^***^	13.73^***^	39.96^***^	40.20^***^				
Moderate mediation index		SE		95% CI				
−0.04		0.01		[−0.06, −0.02]				

a *0 = female; 1 = male*.

b *Reference group is age. Above 46 years as (0, 0, 0,), below 25 as (1, 0, 0), 26–35 as (0, 1, 0), and 36–45 as (0, 0, 1)*.

c *Reference group is educational level. Postgraduate or higher as (0, 0), below a college degree as (1, 0), and undergraduate as (0, 1)*.

* *p < 0.05;*

** *p < 0.01; and*

*** *p < 0.001*.

### The moderating role of psychological detachment

**Figure 2:**
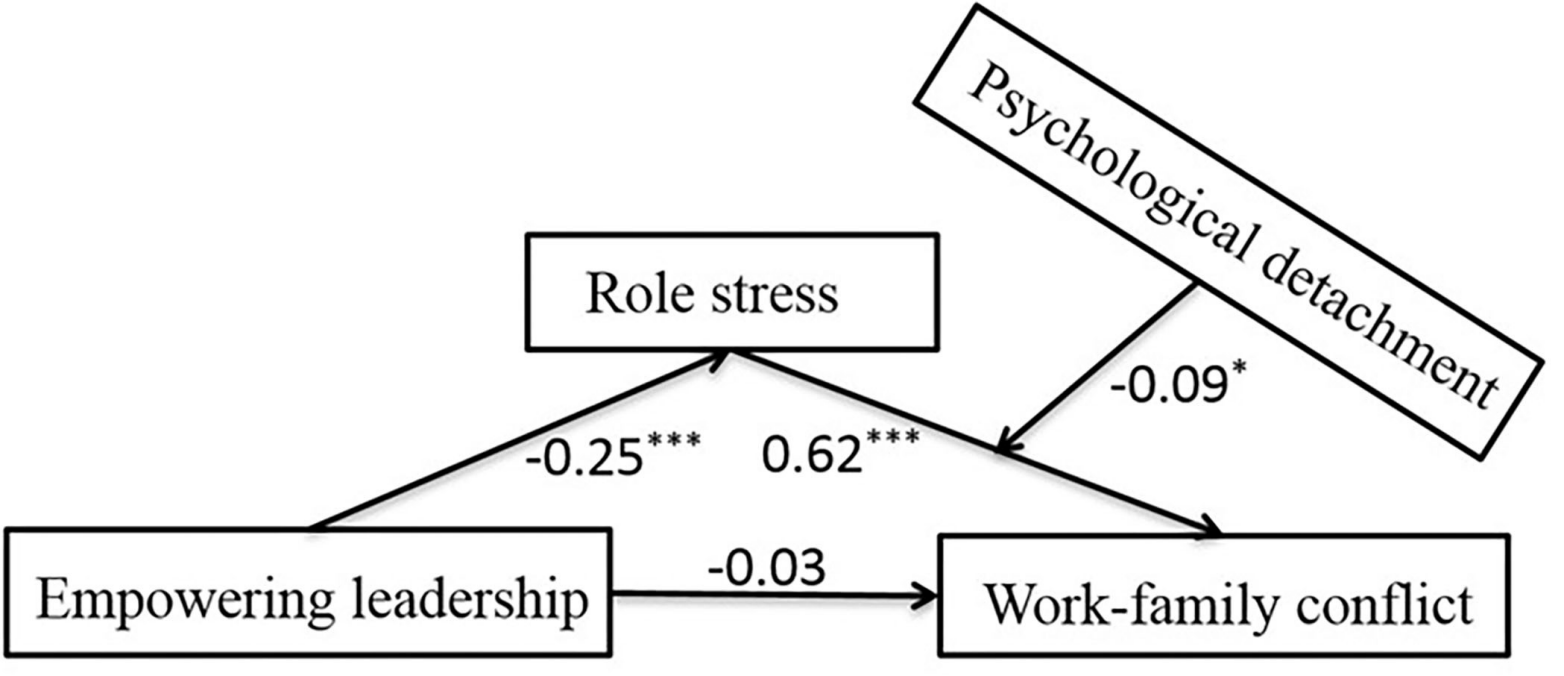
Path coefficients of the moderated meditation model. ***p < 0.001, *p < 0.05.

SPSS PROCESS-Model 14 was used to examine the moderating role of psychological detachment (Hayes, 2013). Overall testing models are shown in Figure 2, and the specific indirect effects are shown in Table 3. The analyses established a conditional indirect effect when the interactions between role stress and psychological detachment were significant, and the bootstrapping confidence intervals did not contain zero. In Figure 2 and Table 3, psychological detachment significantly moderated the indirect effect of empowering leadership on work–family conflict *via* role stress. These results revealed that role stress was negatively associated with work–family conflict (*β* = 0.61, *p* < 0.001), and the product (interaction term) of role stress and psychological detachment had a significant role on work–family conflict [Model 4: *B* = 0.16, 95% CI (0.06, 0.26), SE = 0.04, *t* = 3.21, *p* < 0.001]. Moreover, the direct effect of empowering leadership on work–family conflict was −0.20 [(Model 2: SE = 0.02, 95% CI = (−0.24, −0.15), *p* < 0.001], and the index of the moderated mediation was −0.04 [SE = 0.01, 95% CI = (−0.06, −0.02), *p* < 0.001].

**Figure 3:**
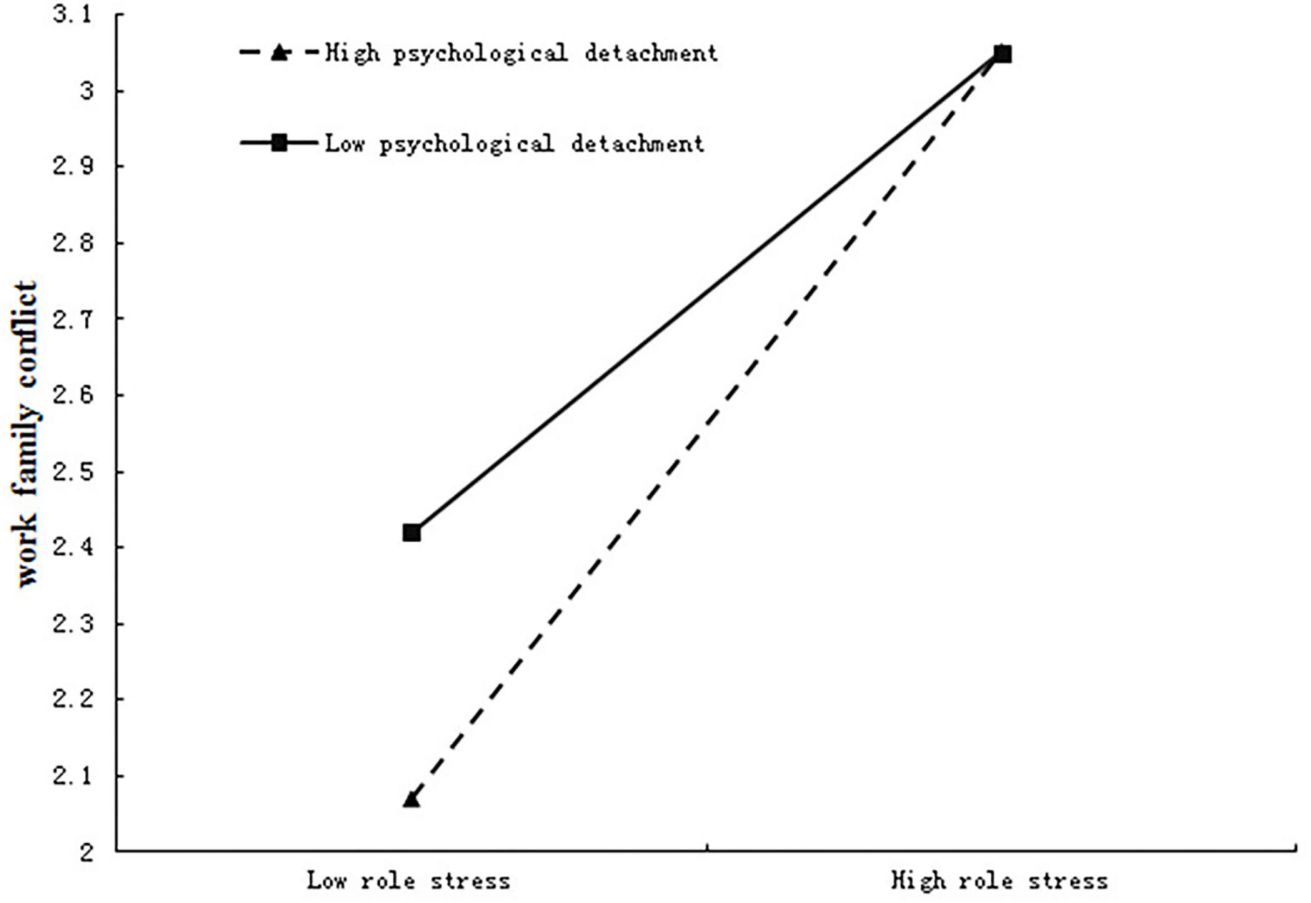
Moderating effect of psychological disengagement between role stress and work–family conflict.

To further test the moderating role of psychological detachment in the relationship between role stress and work–family conflict, psychological detachment was divided into high and low groups by adding or subtracting a standard deviation from the mean and then conducting a simple slope test (see Figure 3).

The results revealed that, under the different conditions of psychological detachment (high, medium, and low), role stress was significantly correlated with work–family conflict (*β* = 0.50, *t* = 9.33, *p* < 0.001; *β* = 64, *t* = 16.97, *p* < 0.001; *β* = 0.78, *t* = 12.83, *p* < 0.001, respectively). In addition, bias-corrected percentile bootstrap analyses further revealed that the indirect effect of empowering leadership on work–family conflict *via* role stress was moderated by psychological detachment. Specifically, for the medical workers who expressed high, medium, and low levels of psychological detachment, the conditional indirect effect between empowering leadership and work–family conflict was significant [effect = −0.19, 95% CI = (−0.23, −0.16); effect = −0.15, 95% CI = (−0.19, −0.13); effect = −0.12, 95% CI = (−0.15, −0.09), respectively; Table 4]. Thus, the results showed that psychological detachment moderated the relationship between empowering leadership and work–family conflict *via* role stress, supporting Hypothesis 3.

**Table 4:** Conditional indirect effect of psychological detachment when role stress was mediated between empowering leadership and work–family conflict.

**Mediator**	**Psychological detachment**	**Effect**	***SE***	***Z***	**95% CI**
Role stress	M-SD	−0.12	0.02	2.09	[−0.15, −0.09]
	M	−0.15	0.01	2.97	[−0.19, −0.13]
	M+SD	−0.19	0.02	3.86	[−0.23, −0.16]

## Discussion

This study principally tested the influence path of empowering leadership on work–family conflict among medical workers, as well as the role of role stress and psychological detachment in the relationship between empowering leadership and work–family conflict. The results revealed that empowering leadership was negatively correlated with work–family conflict, and role stress completely mediated the negative relationship between empowering leadership and work–family conflict. Moreover, the relationship between role stress and work–family conflict was moderated by psychological detachment *via* the latter half path of role stress mediation, finally breaking the leadership “black box” of negative effects and boundaries.

### Theoretical implications

First, this paper explored the negative effect of empowering leadership, its mechanism, boundaries, and its impact on work–family conflict, as well as the mediating effect of role stress and the moderating effect of psychological detachment. It thus contributes to the literature on the effects of empowering leadership on work–family conflict. The results revealed that empowering leadership has a negative effect, and role stress could mediate the impact of empowering leadership on work–family conflict, in which psychological detachment plays a buffering role.

Second, this study broadened the research horizon of the influence effect of empowering leadership, and this conclusion confirmed the concern of some researchers that empowering leadership may bring potentially negative effects while leading to positive results (Amundsen and Martinsen, 2014; Wong and Giessner, 2018). This study confirmed the above conclusions from the perspective of empirical research and thus expands understanding of the negative effects of empowering leadership. This paradigm provides a unique perspective to explore leadership effectiveness, which can help researchers evaluate the role of leadership style more comprehensively (Wang et al., 2019). Notably, a number of recent studies have gradually begun to pay attention to the potential negative impact of empowering leadership (Wong and Giessner, 2018; Chen et al., 2020b), finding that the matching of empowering leadership and subordinate self-leadership had an impact on subordinate role conflict, emotional exhaustion, and job performance. Furthermore, the study has argued that empowering leadership had a negative impact on subordinates by affecting tension and compulsive passions (Hao et al., 2018). This paper explored the negative effects of empowering leadership and showed the impact of empowering leadership on work–family conflict through role theory, which is an important supplement to previous empirical research. In addition, previous studies on the negative impact of empowering leadership were mostly focused on the field of work. For example, Cheong et al. (2016) discussed the adverse impact of empowering leadership on employee work performance, while Hao et al. (2018) found that empowering leadership ignores the cross-domain interpersonal impact of this leadership style. This paper broadened the horizon to intimate family relationships and responds to the call of work–family literature (Lin and Ling, 2016; Hao et al., 2018; Wong and Giessner, 2018; Chen et al., 2020a).

Third, due to the lack of in-depth research into the mechanism underpinning the negative effect of empowering leadership, this paper took the work–family conflict of medical workers as the target population for examining the internal mechanism of the negative effect of empowering leadership from the perspective of role theory, providing new ideas for in-depth understanding of the boundary conditions of the role of empowering leadership. It is conducive with a more comprehensive analysis of the mechanism of empowering leadership, which in turn is conducive to the enrichment and deepening of leadership theory. A number of previous studies have considered role theory for explaining negative effects on employees during work. For example, the research of Lin and Ling (2016) showed that role stress reduces individual job satisfaction and significantly reduces individual wellbeing. Employees' role ambiguity has a significant negative impact on job satisfaction. However, the role pressure or role overload of employees will spill over to the family. Therefore, the problems caused by empowering leadership may cause problems for employees' families, that is, work–family conflict (Wang and Sun, 2019). This idea has lacked empirical support, which the present study has responded to. Through empirical research, this paper discusses the work–family conflict caused by empowering leadership through role pressure, demonstrating the impact of employee work pressure on the family, and provides a theoretical analysis for how empowering leadership can play a better role.

Finally, by incorporating psychological detachment into the research framework, it was found that psychological detachment could moderate the negative influence of empowering leadership on work–family conflict through role stress. As Xu (2016) pointed out, from the perspective of responsible research, a future study should focus on mining the boundary conditions of the negative effects of positive leadership, especially the factors that can avoid these negative effects. Psychological detachment is an important strategy of resource management and an effective means of halting the continuous loss of resources, in turn saving resources for the next period of work (Sonnentag and Fritz, 2015; Johannes and Andrea, 2017; Ma et al., 2021). According to a previous summary of the internal mechanism driving the negative effects of empowering leadership, the role conflict of employees could be the negative outcome of empowering leadership. Therefore, the factors that can help employees reduce the role stress caused by empowering can provide a more sufficient guarantee of the effectiveness of empowering leadership (Lee et al., 2017; Lin and Luo, 2017; Hao et al., 2018; Wong and Giessner, 2018; Yin and Xing, 2018; Wang and Sun, 2019; Chen et al., 2020a; Jiang and Xu, 2020; Song and Chen, 2021). Psychological detachment is an important buffer in response to this request, which enables employees in stressful situations to free themselves from pressure, especially after returning home. Employees can eschew the excessive requirements of empowering leaders, stop attending to work, and focus on their family. This helps employees with their family-based relationships, better balancing their relationship between work and family (Shi and Zhen, 2021; Wang et al., 2021). Thus, this paper focused on breaking the “black box” of the negative effect of leadership and its mechanism, exploring the boundary conditions of the negative effect of positive leadership, and in particular exploring the factors that can avoid these negative effects. Compared with previous research, this paper clarified those factors that can weaken or avoid the negative effect of positive leadership, thus having important theoretical value and epochal significance for guiding enterprises to use leadership scientifically in practice.

### Practical implications

This paper has significance for management practices. In the post-pandemic era, with the uncertainty, variability, and severity of the pandemic, and after the brutality of the pandemic experienced by those fighting it on the frontline, extreme events for medical workers and in the relationships between those workers and their leaders occur frequently. Leadership and good working conditions for medical workers are scarce resources, and maintaining them is vital. First, empowering leadership can produce positive effects in specific practice, but also has a potentially negative impact, affecting the work–family conflict of employees. Therefore, in management practices, leaders should fully realize the negative effect that may occur from authorizing their workers to be more independent, and so leaders should be prepared for thinking and behavior. Second, after authorizing the workers, work–family conflict is mainly caused by role stress. Therefore, leaders should communicate with employees in a timely fashion to confirm whether the empowering responsibilities conflict with the original responsibilities of the employees, or whether the requirements are being clearly communicated to the employees to ensure that the role is clear, so as to make the employees fully understand the requirements of the role and in turn be able to efficiently complete the task (Kuhnel et al., 2012). Otherwise, leaders should ascertain whether they need not call the employees outside of working hours about work, or ensure that the employees take a holiday at appropriate times to relax and restore their resources for work. In addition, employees themselves need to balance the relationship between work and non-work. The results of this paper suggest that psychological detachment can buffer the work–family conflict caused by role stress. Therefore, employees should be encouraged to distinguish between periods of work and non-work, to be efficient at completing their tasks during periods of work, and to be family-focused after work so as not to further deplete their resources. Furthermore, a harmonious relationship between work and family should be promoted. All these initiatives can achieve a “win–win situation.” Therefore, based on painful experiences, multiple stresses, and life and death scenarios within the context of the COVID-19 pandemic, first, the medical workers themselves should be encouraged to use various ways to avoid family–work conflicts and to communicate in a timely fashion with their leaders, reducing the generation of negative emotions and enhancing positive cognition, effectively using emotional intelligence to enhance their internal strength and external resources. Second, the medical workers should avoid exacerbating the stresses that they experience which in turn depletes their psychological resources and instead direct more attention to the families of medical workers, helping to resolve their needs where necessary.

### Limitations and future research

Although this paper tested the study hypotheses and generated a deeper understanding of the relationships between empowering leadership and work–family conflict, thus breaking open the leadership “black box” of negative effects and boundaries, the study has some limitations. First, from the perspective of role theory, this paper verified the impact of empowering leadership on work–family conflict. In future, researchers should consider discussing the mechanism from other theoretical perspectives. For example, the relationship between the two could be studied from the perspective of matching theory. When leaders do not want to show an empowering leadership style, this style of leadership will reduce the work performance of subordinates or cause work to interfere with the family (Liden et al., 2014). Second, this paper suggested that psychological detachment as a factor to weaken or avoid the negative impact of empowering leadership on work–family conflict is an important finding.

Psychological detachment, as an individual's behavior, can play a role in alleviating negative effects. However, in organizations, leaders and subordinates are more likely to weaken this adverse effect through institutional design or atmosphere construction. Therefore, future research, while continuing to explore individual factors to alleviate negative effects, should focus on mining from the organizational level. Of course, the relationship between empowering leadership and work–family conflict is very complex, and there are other mediating and moderating variables yet to be examined. For example, a preview study showed that emotional intelligence was related to stress and work–family conflict, such as work engagement (Brunetto et al., 2012). As a psychological trait, emotional intelligence is an effective psychological resource to cope with stresses and negative situations, which may aid individuals in relieving stresses and decrease the consumption of their internal resources (Karim and Weisz, 2011). People with high emotional intelligence may experience less stress than people with low emotional intelligence (Kalyoncu et al., 2012). This difference indicates that emotional intelligence may buffer the negative relationship between empowering leadership and work–family conflict. Thus, future research may also investigate the roles of other variables to better describe the mechanisms between empowering leadership and work–family conflict.

Finally, this study used one-time sampling for data collection, and the subjects were mainly medical workers, which generated common method bias effects and external validity promotion restrictions. However, this study used large samples and found no serious common method deviation problems. Because the cross-sectional nature of this paper could not verify causality between the variables, longitudinal tracking, work logs, experiments, and other research designs should be adopted to avoid similar problems through multi-point, one-to-one paired sampling (such as the managers, colleagues, and the relative family members), to reveal causal relationships. For example, Liao et al. (2021) used empirical sampling to explore the fluctuation of daily public servant leadership behavior and its negative impact on leaders. This study provides reference and guidance for exploring the negative effects of empowering leadership by using the fluctuation research method. In addition, to improve the external validity of this study, future work should expand the source of the sample by sampling from other groups, to further improve the external validity, explore the role of cultural differences, and carry out a cross-cultural study to verify the model presented in this paper.

## Data availability statement

The original contributions presented in the study are included in the article/supplementary material, further inquiries can be directed to the corresponding author.

## Ethics statement

This study involved human participants and was reviewed and approved by the morality and Ethics Committee of the Public Course Teaching Department of Shandong University of Science and Technology. The participants provided oral informed consent to participate in this study.

## Author contributions

HZ, XS, and LF wrote the papers. KS and RL provided the whole idea. All authors contributed to the article and approved the submitted version.

## Funding

This study was supported by Humanities and Social Sciences Project of Shandong Province in 2021 (2021-YYJY-07) and Shandong University of Science and Technology Talent Introduction Scientific Research Startup Fund Project (2017RCJJ082).

## Conflict of interest

Author XS was employed by ZR Holdings Limited. The remaining authors declare that the research was conducted in the absence of any commercial or financial relationships that could be construed as a potential conflict of interest.

## Publisher's note

All claims expressed in this article are solely those of the authors and do not necessarily represent those of their affiliated organizations, or those of the publisher, the editors and the reviewers. Any product that may be evaluated in this article, or claim that may be made by its manufacturer, is not guaranteed or endorsed by the publisher.
